# Biology and Interaction of the Natural Occurrence of Distinct Monopartite Begomoviruses Associated With Satellites in *Capsicum annum* From India

**DOI:** 10.3389/fmicb.2020.512957

**Published:** 2020-10-07

**Authors:** Megha Mishra, Rakesh Kumar Verma, Avinash Marwal, Pradeep Sharma, R. K. Gaur

**Affiliations:** ^1^Department of Biosciences, School of Liberal Arts and Sciences, Mody University of Science and Technology, Sikar, India; ^2^Department of Biotechnology, Mohanlal Sukhadia University, Udaipur, India; ^3^Biotechnology Unit, ICAR-Indian Institute of Wheat and Barley Research, Karnal, India; ^4^Department of Biotechnology, D.D.U Gorakhpur University, Gorakhpur, India

**Keywords:** *Capsicum annum*, mixed infection, satellites, sequence variability, recombination

## Abstract

Chili (*Capsicum annuum* L.) is an important vegetable and spice crop of tropical and sub-tropical regions. Chili plants showing upward leaf curling, leaf crinkling, and leaf yellowing symptoms, collected from Sikar district of Rajasthan, India, were found to be associated with begomovirus and satellite molecules. The presence of virus was confirmed by PCR using virus-specific primer. The full-length genomic DNA-A of three begomovirus (MM-1, CS-1 and RV-1) and two satellites (MM-2 and MM-3) were cloned which was identified from single symptomatic chili plant. The genome organization of isolated three viruses is similar to those of other Old World monopartite begomoviruses. The comparison of the sequences and closest phylogenetic relationships for the begomoviruses, betasatellite and alphasatellite DNAs revealed that MM-1 was designated as DNA-A of *Chili leaf curl virus* (ChiLCV), CS-1 is considered to be a new distinct species of *Tomato leaf curl Gujrat virus* (ToLCGV) whereas RV-1 as a new strain of *Cotton leaf curl Multan virus* (CLCuMuV). The DNA-A component of ChiLCV showed 8.6%, ToLCGV of 16.6% and CLCuMuV of 7.7% average evolutionary divergence, concomitantly, the betasatellite and alphasatellite molecule had 9.9% and 5.9% overall sequence divergence, respectively. Interestingly, most of the begomoviruses were found to be intra-species recombinants. The dN/dS ratio and Tajima *D* value of all viral DNA-A component and their associated betasatellite showed their selective control on evolutionary relationships. The nucleotide substitution rates were determined for the DNA-A genomes of ChiLCV (7.22 × 10^–4^ substitutions site^–1^ year^–1^), CLCuMuV (1.49 × 10^–4^ substitutions site^–1^ year^–1^), ToLCGV (7.47 × 10^–4^ substitutions site^–1^ year^–1^), the genome of associated ChiLCB (4.20 × 10^–4^ substitutions site^–1^ year^–1^) and CLCuMuA (1.49 × 10^–4^ substitutions site^–1^ year^–1^). Agro-inoculation studies indicate that the presence of DNA betasatellite induce severe symptoms in *N. benthamiana* and chili, suggesting prerequisite association for typical disease development.

## Introduction

Geminiviruses are critical plant DNA viruses infecting a variety of crops, including both monocotyledonous and dicotyledonous plants (Kumar, 2019). A study examining genome-wide pairwise sequence identity, genome organization, host range, and insect vector, classified the family *Geminivirade* into nine genera: *Becurtovirus, Begomovirus, Capulavirus, Curtovirus, Eragrovirus, Grablovirus, Mastrevirus, Topocuvirus, and Turncurtovirus* (Zerbini et al., 2017; Varsani et al., 2017; Gnanasekaran et al., 2019). *Begomoviruses* as one of the principal genus of the family *Geminiviridae* encompasses more than 320 diverse species prevailing in the tropical to the subtropical vicinity of the old and new world (Hanley-Bowdoin et al., 2013; Brown et al., 2015), and are transmitted by the vector whitefly, *Bemisia tabaci* (De Barro et al., 2011). Begomovirus genome is made up of either single circular (monopartite; DNA-A) or two separate circular DNA components (Bipartite; DNA-A/DNA-B) of ∼2.5–2.7 kb each, encoding five to seven proteins which are implicated in viral replication, movement, transmission, and pathogenesis (Ito et al., 2009; Roy et al., 2019).

The DNA-A component encodes for AV1 and AV2 ORFs are present on the virion sense strand and encodes coat protein (CP) and movement protein (MP) respectively, complementary sense strand has AC1, AC2, AC3, and AC4 ORFs encoding replication associated protein (Rep), transcriptional activator protein (TrAP), replication enhancer protein (REn) and AC4 protein, respectively (Zerbini et al., 2017). DNA satellites associated with monopartite begomoviruses have been described as alphasatellite, betasatellite, and deltasatellitets (Fiallo-Olivé et al., 2016) forming disease complexes and thus emerged as a serious threat to agriculture globally (Rodríguez-Negrete et al., 2019). Alphasatellite belongs to the *Geminialphasatellitinae* subfamily, a member of the family *Alphasatellitidae*, comprising a single gene encoding alpha-rep protein in the virion-sense, and a hairpin structure at their origin of replication (Briddon et al., 2018). Likewise, betasatellites having a size of ∼1.3 kb are pathogenicity-determinant molecules, a suppression of both transcriptional and post-transcriptional gene silencing, enhance disease severity in plants through stifling its host defense activities (Briddon et al., 2008; Patil and Fauquet, 2010) via its single gene βC1 product. Betasatellites also contain a region of sequence rich in adenine and a ∼150 nt region, known as the satellite conserved region (SCR) that is highly conserved between all betasatellites (Briddon et al., 2003; Zhou, 2013). The SCR contains a predicted hairpin structure within the loop a nona-nucleotide sequence (TAATATTAC) that for geminivirus DNA replication. Earlier finding, suggested that the alphasstellite play an important role in the epidemiology of begomovirus/betasatellite complex (Xie et al., 2010).

Chili is known due to its active ingredient Capsaicin. It is an excellent source of Calcium and Vitamin C, while studies showed its anti-proliferative effect against cancer sinusitis and bronchitis etc. In India, Chili is cultivated almost in every state, including Andhra Pradesh, Karnataka, Rajasthan, Tamil Nadu, Maharashtra, Orissa, and West Bengal. In India, during 2017-18, green chilies were cultivated in 309000 hectares with a total production of 3592000 metric tonnes (Horticultural Statistics at a Glance, 2018). Leaf Curl disease of chili has been reported by several researchers worldwide. Chili infection by viruses is a serious threat to chili production and causes significant destruction of chili plant and yield loss (King and Adams, 2011). Nearly 100 percent loss of marketable fruit has been reported (Kumar S. et al., 2011; Kumar Y. et al., 2011; Senanayake et al., 2012). Sixty five (65) plant viruses are so far reported to infect chili plant worldwide (Devi and Devi, 2020). In India, eleven viruses have been reported to occur naturally on chili (Zehra et al., 2017) including ChiLCV, *Cucumber mosaic virus* (CMV), *Pepper venial mottle virus* (PVMV), *Tobacco leaf curl virus* (TLCV), *Potato virus X* (PVX), *Potato virus Y* (PVY), *Tobacco ring spot virus* (TRSV), *Pepper vein bending virus* (PVBV), *Tomato leaf curl New Delhi virus* (ToLCNDV), *Chili mosaic virus* and *Capsicum chlorosis virus.*

Mixed infection of *Tomato leaf curl virus* and ChiLCV in chili plant was also previously reported in India (Khan et al., 2006), however, there is no report on the association of CLCuMuV with *Tomato leaf curl Gujrat virus* (ToLCGV) and ChiLCV in Chili.

Emergence of new variants of viruses due to recombination and mutations in the genomes, introduction of susceptible plant varieties and change in climatic conditions are responsible aggravating the increasing incidence and disease severity problems of begomovirus during the last two decades (Juárez et al., 2019). In India, due to wider agro-climatic conditions and mixed crop patterns supporting the year-round survival of the whitefly vector responsible for widening and overlapping the host range of begomoviruses as reported earlier (Tripathi and Verma, 2017; Malathi et al., 2017). Incidence of mixed infections with different begomoviruses and satellite molecules as well as the emergence of the new variants of viruses due to recombination of existing ones (Fiallo-Olivé et al., 2019), has manifested the adaptability to new hosts, which has posed such a serious threat to many economically important crops (Varma and Malathi, 2003). Recombination is a vital evolutionary approach for viruses (Pagán and García-Arenal, 2018; Zaffalon et al., 2012) to get adapted to new environmental conditions (Verma et al., 2015) concomitantly, it is the major driving force in the evolution of many viruses and also has been adequately revealed for ssDNA viruses especially begomoviruses (Martin et al., 2011).

In this study, we have identified and characterized three distinct begomoviruses and association of alphasatellite and betasatellite with chili plant showing leaf curl symptoms from Sikar, India. We further, analyzed the sequence variability, phylogenetic relationship, and recombination breakpoints in the genome sequences of ChiLCD.

## Materials and Methods

### Sample Collection and DNA Extraction

**FIGURE 1:**
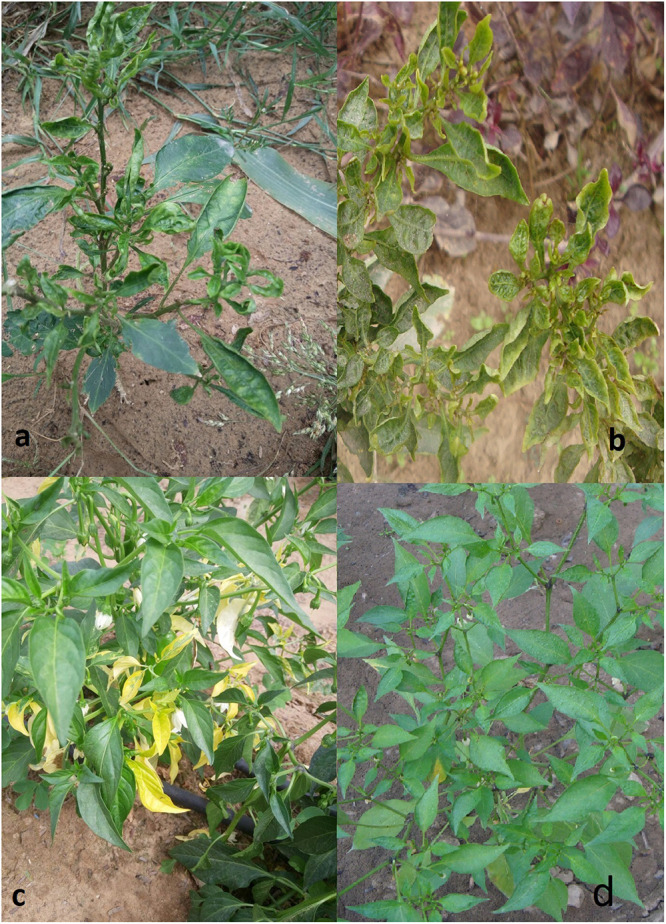
Typical symptoms observed in the chili field in Sikar, Rajasthan, India. The common symptoms that appeared on leaves were **(a)** leaf curling, **(b)** leaf crinkling, **(c)** leaf yellowing, and **(d)** healthy.

During the routine survey (2017-18) for begomovirus infection, chili plants showing upward leaf curling, leaf crinkling, and leaf yellowing symptoms (Figure 1) were collected from Sikar district of Rajasthan, India. To investigate the potential begomovirus infection, total DNA was extracted from leaves of infected plants and healthy plants using the CTAB method (Sahu et al., 2018). The quality and quantity of extracted DNA was subsequently analyzed using 0.8% agarose gel and spectrophotometer values.

### Sequencing and Alignment Analysis

In order to confirm the presence of begomovirus infection, polymerase chain reaction (PCR) was carried out using CP gene-specific degenerate primers AC-1048 (5′GGRTTDGARGCATGHGTACATG3′) and AV-494 (5′GCC YATRTAYAGRAAG CCMAG3′) (Wyatt, 1996). Beta01 (5′-GGTACCACTACGCTACGCAGCAGCC-3′) and beta02 (5′-GG TACCTACCCTCCCAGGGGTACAC-3′) primer set was used for the detection of betasatellite (Briddon et al., 2002) and subsequently, primer set UN101 (5′-AAGCTTGCGACTAT TGTATGAAAGAGG-3′)/UN102 (5′-AAGCTTCGTCTGTCTT AC GAGCTCGCTG-3′) was employed to amplify the alphasatellite (Bull et al., 2003). To obtain the complete genome sequences, rolling circle amplification technology (RCA) was performed by using the TempliPhi 100 Amplification Kit (GE Healthcare, Life Sciences, United States). The RCA product as digested to linearize the genome using single-cutting restriction endonucleases *Hin*dIII (for ChiLCV and ToLCGV), *Bam*HI (CLCuMuV and CLCuMuA) and *Kpn*I (for ChiLCB). The resultant products of ∼2.7 and∼1.3 kb, were eluted and cloned into pUC19 vector (Fermentas, Arlington, Canada) and designated as M1 (ChiLCV), CS-1(ToLCGV), RV-1(CLCuMuV), MM2 (ChiLCB) and MM3 (CLCuMuA) (Table 1). To verify the identity of the isolate, the clones were sequenced and analyzed using restriction enzymes cut at unique restriction sites. Besides the sequences determined in this study, nearly twenty-five (25) reference sequences for each begomovirus and satellites were retrieved from NCBI database for further analysis. The DNA sequence was determined for each of five putative full-length begomoviral genomic and satellites clones and analyzed using BLASTn algorithm to query the GeneBank database (NCBI) (Supplementary Tables 1–5). The open reading frames (ORFs) were identified using ORF finder software^1^. The percentage pairwise nucleotide sequence identities of cloned begomoviruses and associated DNA-satellites were generated by MUSCLE based alignment in SDT v.1.21 (Muhire et al., 2014).

**TABLE 1:** Features of begomoviral DNA-A and associated satellites clones obtained in this study.

**Begomovirus genome/DNA-A molecule**								
Clone name*	**size (nt)**	**Coding sequences [coordinates/coding capacity (number of amino acids)/predicted molecular mass (kDa)]**						
		**AV1 (CP)**	**AV2 (pre-CP)**	**AC1 (Rep)**	**AC2 (Trap)**	**AC3 (Ren)**	**AC4**	**AC5**
MM1	2761 bp	309-1082/257/29.75	149-514/121/13.91	1531-2616/361/40.08	1224-1628/134/15.30	1079-1483/134/15.63	2166-2459/97/10.81	–
CS1	2757 bp	304-1074/256/29.83	144-491/115/13.22	1523-2608/361/40.75	1216-1620/134/15.19	1071-1475/134/15.84	2158-2451/97/10.78	–
RV1	2754 bp	292-1062/256/29.61	132-488/118/13.80	1511-2599/362/40.67	1162-1614/150/17.39	1065-1469/134/15.66	2143-2445/100/11.28	76-807/243/27.22

**Betasatellites/Alphasatellite molecule**								
		**βC1**	**Alpha rep**					

MM-2	1389 bp	201-563/120/14.06	–					
MM-3	1366 bp	–	77-1024/315/36.78					

* Note- MM1, ChiLCV (MF737343); CS1, ToLCGV (MF737344); RV1, CLCuMuV(MF737345); MM2, ChiLCB (MF737346); MM3, CLCuMuA (MF737349).

### Phylogeny and Recombination Analysis

We performed Model Test to identify the best nucleotide substitution model by means of the lowest BIC score and subsequently Maximum-likelihood (ML) phylogenetic tree for each data set was constructed by using MEGA X (Kumar et al., 2018) with 1000 bootstrap value. To detect potential recombination events, aligned nucleotide sequences of different begomovirus isolates were screened by different algorithms implemented in RDP 4.2 (RDP, GENECONV, CHIMERA, BOOTScan, MaxChi, 3Seq, and PhylPro) (Martin et al., 2015) with default parameters and 0.05 highest acceptable Bonferroni corrected p-value. Recombination events detected by at least three out of seven algorithms were considered relevant to avoid false-positive results. Rates of different transitional substitutions, dN (non-synonymous)/dS (synonymous) ratio, Tajima’s neutrality test, transition/transversion bias (R), and average evolutionary divergence were calculated using MEGA X program.

### Population Structure and Substitution Rate Estimation

Genetic variability in the population of viruses was determined by DnaSP v. 6.12 (Rozas et al., 2017). Various parameters viz. total number of mutations (η), average number of nucleotide difference between sequences (k), total number of segregating sites (s), nucleotide diversity (π), Watterson’s estimate of the population mutation rate based on the total number of segregating sites (θ-w) and the total number of mutations (θ-η) were calculated (Lima et al., 2017). To check the mutational bias in the genome of various begomovirus isolates the phylogenetic approach was employed. Per site nucleotide substitution rate and mutation at various codon positions, were estimated by using 10^7^ chain length in the Bayesian Markov Chain Carlo method in BEAST v.1.8.4 (Drummond and Rambaut, 2007). Each dataset was analyzed by both strict and relaxed molecular clock (uncorrelated exponential and uncorrelated lognormal) and best-fit clock and coalescent constant demographic models were identified and achievement of suitable effective sample sizes for these parameters estimated by using Tracer v 1.5^2^.

### Construction of Clones and Agro-Inoculation

Infectivity of the three cloned begomovirus and associated satellites was studied in *N. benthamiana* along with the chili plants using the Agrobacterium-mediated inoculation system (Singh et al., 2012). Dimeric head-to-tail tandem repeats of MM1, CS-1, RV-1, MM2 and MM3 were developed by digested with restriction endonucleases to yield presumed monomeric virus and associated satellites. The MM1 and CS-1 were digested with *Hin*dIII- *Sal*I to release a ∼1000 bp and ligated into binary vector pCAMBIA1304 (Cambia, Canberra, Australia). The full-length insert of both the clone, released using *Hin*dIII, ligated into the binary vector to Chi-A and Tom-A infectious clone. Similar strategy was used for the infectious clone of RV-1 in pCAMBIA1304 using a ∼1000 bp *Bam*HI-*Sal*I fragment and full-length insert using *Bam*HI to produce Cot-A. For, infectious clone of MM2, *Kpn*I-*Xba*I is used for the release of ∼600 bp from the ChiLCB clone and ligated into the binary vector followed by full length insert using *Kpn*I named as Chi-β. Further, a fragment of ∼550 kb was cut from MM3 clone using *Bam*HI and *Eco*RI and cloned into pCAMBIA1304. Then, the complete genome of CLCuMuA was excised from MM3 insert using *Bam*HI and ligated to binary vector to get a binary plasmid Cot-α. All the infectious clone contains the IR to ensure successful infection.

The insertion and orientation were confirmed by restriction digestion with appropriate enzymes. Recombinant plasmids and empty vector were introduced into *Agrobacterium tumefaciens* LB 4401 by electroporation with a Gene Pulser apparatus (Eppendorf, United States). Agro-inoculation was performed as described by Sahu et al. (2015). Different combinations of clones i.e., Chi-A, Chi-A + Chi-β, Chi-A + Cot-α, Chi-A + Chi-β + Cot-α, Tom-A, Tom-A + Chi-β, Tom-A + Cot-α, Tom-A + Chi-β + Cot-α, Cot-A, Cot-A + Chi-β, Cot-A + Cot-α, Cot-A + Chi-β + Cot-α, Chi-A + Tom-A + Cot-A + Chi-β + Cot-α and empty pCAMBIA1304 as negative control were introduced to check the efficiency of inoculation. Agro-inoculation was performed in *N. benthimiana* plants. The inoculated plants were incubated in a growth chamber with 16h day light at 25 ± 2°C. For confirmation, DNA blots were hybridized with α^32^P-labeled dCTP coat protein gene (AV1) of previously mentioned begomoviruses and betasatellite and alphasatellite by nick translation. Viral DNA blots were detected using a phosphor image analyzer.

## Results

### Genome Organization of Three Begomovirus Species ChilCD

To identify the causal begomovirus infection in symptomatic chili plants, using PCR based amplification of ∼560 bp fragment with universal primers confirm the presence of begomoviral (Wyatt and Brown, 1996) and associated betasatellite (Briddon et al., 2002; Bull et al., 2003) components, but amplification was not optimal with the presence of non-specific amplicons. Therefore, RCA mediated enrichment strategy to obtain specific amplification yielded DNA fragments of either ∼2.7 kb or sub-genomic amplicon of ∼1.3 kb, were used. The monopartite begomoviral genomes were cloned from the RCA-amplified products obtained using total DNA that was extracted from chili plants collected from Sikar, Rajasthan. We obtained complete nucleotide sequences of the MM1, RVI, CS1 isolate consisted of 2761, 2754 and 2757 nucleotides, respectively (accession numbers, MF737343, MF737345, and MF737344). The genome organizations of these viruses were like those of Old World begomoviruses constructed by using PlasMapper tool (Xiaoli et al., 2004) (Supplementary Figure 1). Sequence alignment followed by pairwise comparisons revealed that the three isolates shared 78–76% nt sequence identity. Inspection of these genomic sequences revealed that they had features like other monopartite begomoviral genomes, based on the size and the characteristic organization of the six ORFs (AV1, AV2, AC1, AC2, AC3, and AC4) and a conserved nona-nucleotide sequence TAATATTAC, required for transcription and viral replication (Table 1) (Eagle and Hanley-Bowdoin, 1997). PCR amplification to detect DNA-B components associated with MM1, CS1 and RV1 using primers PCRc1 and PBL1v2040 (Bandaranayake et al., 2016), were unsuccessful. Thus, we concluded that MM1, CS1 and RV1 are monopartite begomoviruses. MM2 (MF737346) consisted of 1389 nucleotide and had a structural feature like betasatellite i.e., single ORF βC1, satellite conserved region and A-rich region. MM3 (MF737349) was 1366 nucleotide and had three conserved domains, hairpin structure, alpha-rep and A-rich region (Supplementary Figure 1).

Based on nucleotide sequence identity (91%) as the species demarcation threshold for DNA-A (Brown et al., 2015), these isolates were designated into two distinct species, including one newly identified species of ToLCGV. The genome of MM1 shared the highest identity (96%) with ChiLCV isolate T-31 (HE806437) from Oman with 100% query cover. While ToLCGV isolate CS-1 exhibited a maximum identity of 89% with isolate C1 of ToLCGV (KP725055). Thus, isolate CS1 was considered as a new begomovirus species suggested by ICTV guidelines (Brown et al., 2015). The RV1 isolate shared utmost identity (91%) with CLCuMuV isolate Mohanpura Rajasthan (KC412251) considered to be a new strain designated as *Cotton leaf curl Multan virus-Chili* (CLCuMuV-Chili).

Likewise, MM2 exhibited the highest sequence identity 94% with clone chM13 Pakistani isolate (AM279661) therefore, designated as *Chili leaf curl betasatellite* (ChiLCB) and MM3 isolate sowed similarity with AR4 isolate (LN831966; 90%) from Pakistan therefore, designated as *Cotton leaf curl Multan alphasatellite* (CLCuMuA) (Supplementary Figure 2). The nucleotide sequences of the ORFs of the three begomoviruses and associated satellite DNA were also compared to those of other Begomoviruses (Table 2).

**TABLE 2:** Highest Percent identity (nucleotide) for full-length, ORFs of begomovirus and associated satellites clones obtained from chili plant.

**Begomovirus Component**	**MM1**	**CS-1**	**RV-1**	**MM-2**	**MM-3**
DNA-A	95.76 (HE806437) ChiLCV[OM:11]	89.21 (KP725055) ToLCGV[IN:14]	91.03 (KC412251) CLCuMuV[IN:05]	94.17 (AM279661) ChiLCB[PK:04]	90.25 (LN831966) CLCuMuA[PK:15]
AV1 (CP)	97.93 (KF229718) ChiLCV[OM:12]	89.91 (KP725055) ToLCGV[IN:14]	95.19 (KJ488991) AEV[IN:12]	–	–
AV2 (pre–CP)	99.73 (KJ649706) ChiLCV[IN:10]	100 (KY026598) PaLCuV[IN:15]	100 (KC412251) CLCuMuV[IN:05]	–	–
AC1 (Rep)	96.13 (MK757216) ChiLCV[OM:16]	90.33 (KP725055) ToLCGV[IN:14]	93.50 (KC412251) CLCuMuV[IN:05]	–	–
AC2 (Trap)	98.27 (HM007104) ChiLCV [IN:09]	91.48 (MF184923) CLCuMuV[IN:17]	93.32 (AM691745) PepLCLV[PK:07]	–	–
AC3 (Ren)	95.06 (HE806437) ChiLCV[OM:11]	95.87 (MG373556) CLCuMuV[IN:17]	95.63 (AM691745) PepLCLV[PK:07]	–	–
AC4 (Possible symptom determinant)	99.32 (KP698316) ToLCGV[IN:14]	85.87 (MK087120) PaLCuV[IN:18]	88.12 (GU111996) OELCuV[IN:06]	–	–
AC5 (pathogenicity determinant)	–	–	95.63 (KC412251) CLCuMuV[IN:05]	–	–
βC1	–	–	–	97.80 (KJ700655) ChiLCB[IN:14]	–
Rep	–	–	–	–	92.51 (FR772085) AConSLA [PK:06]

* Note- MM1, ChiLCV (MF737343); CS1, ToLCGV(MF737344); RV1, CLCuMuV(MF737345); MM2, ChiLCB(MF737346); MM3, CLCuMuA (MF737349).

The predicted nucleotide sequences of the ORFs (AV1, AV2, AC1, AC2, AC3, AC4, and AC5) of all the begomovirus isolates and associated satellite molecules were also compared with other begomoviruses (Table 2). The ORFs of ChiLCV (MF737343) showed maximum nucleotide ORF similarity (95.06– 99.73%) with other geographical isolates of ChiLCV and ToLCV reported from India and Oman. However, the ORFs of ToLCGV (MF737344) were observed to be maximum identical (85.87– 100%) with various Indian isolates of ToLCV, CLCuMuV and *Papaya leaf curl virus* (PaLCuV). The coding nucleotide sequence of the CLCuMuV (MF737345) shared 88.12– 100% similarity with various isolates of *Okra enation leaf curl virus* (GU111996), *Pepper leaf curl Lahore virus* (AM691745), *Ageratum enation virus* (KJ488991) and CLCuMuV (KC412251) (Table 2). The maximum divergence was observed in AC1, AV1 and AC4 region; signify the strong evidence of recombination in the virus genome. βC1 of ChiLCB shared maximum nt identity with Indian isolate of ChiLCB (KJ000655) whereas, Rep nucleotide sequence of CLCuMuA showed 92.51% similarity with *Ageratum conyzoides* associated symptomless virus alphasatellite (FR772085) from Pakistan. Based on the highest percentage identity, our result showed that there is a frequent recombination and mutation in the sequence of present study.

### Genetic Diversity and Phylogeny

Rates of different transitional substitutions of ChiLCV were ranged from 8.75 to 17.55 and transversional substitutions were ranged from 5.14 to 7.46 and the estimated transition/transversion bias (R) was 0.94. In addition, ToLCGV transition rates ranged from 9.75 to 19.22 and the transversion rates range from 4.79 to 7.19 and the transition/transversion bias (R) was calculated to be 1.08, likewise, the transition rates and transversion rates of CLCuMuV ranged from 13.43 to 15.42, 5.01 to 7.30, respectively and the transition/transversion bias (R) was 1.04. Sequence comparison of DNA-A isolates with other geographical isolates showed average evolutionary divergence ranged from 2.1% to 10.3% (Table 3). Whereas the betasatellite showed a 7.2% overall sequence divergence, likewise alphasatellite exhibited a 3.1% average evolutionary divergence in nucleotide sequences. Pamilo–Bianchi–Li (PBL) method implemented in MEGA X was used to check the pairwise genetic differences at non-synonymous (*d*_N_) and synonymous (*d*_S_) nucleotide positions because the *d*_N_/*d*_S_ ratio can be used as an index representing the pattern of selective constraint in evolutionary relationships (Nei and Gojobori, 1986). The *d*_N_/*d*_S_ ratio of DNA-A of ToLCGV was higher than the ChiLCV and CLCuMuV. The CLCuMuA has a higher value of *d*_N_/*d*_S_ ratio (4.00) as compared to the ChiLCB (Table 3).

**TABLE 3:** Sequence variability analysis in begomoviral and associated satellite clones.

**Clone name**	**Best Model**	**Mean Distance (d)**	**dN**	**dS**	**dN/dS**	**Tajima *D***
MM1	T92	0.021 ± 0.006	0.019 ± 0.002	0.072 ± 0.007	0.2638	− 0.1609
MM2	T92 + G	0.072 ± 0.012	0.072 ± 0.006	0.062 ± 0.008	1.1612	− 0.7305
RV1	HKY + G + I	0.103 ± 0.015	0.111 ± 0.005	0.071 ± 0.007	1.5633	− 0.1161
MM3	T92 + G	0.031 ± 0.005	0.036 ± 0.004	0.009 ± 0.002	4.000	− 1.3182
CS1	TN93 + G	0.037 ± 0.002	0.039 ± 0.002	0.024 ± 0.002	1.625	−1.8906

* Note-MM1, ChiLCV (MF737343); CS1, ToLCGV (MF737344); RV1, CLCuMuV (MF737345); MM2, ChiLCB(MF737346); MM 3, CLCuMuA (MF737349).

**FIGURE 2:**
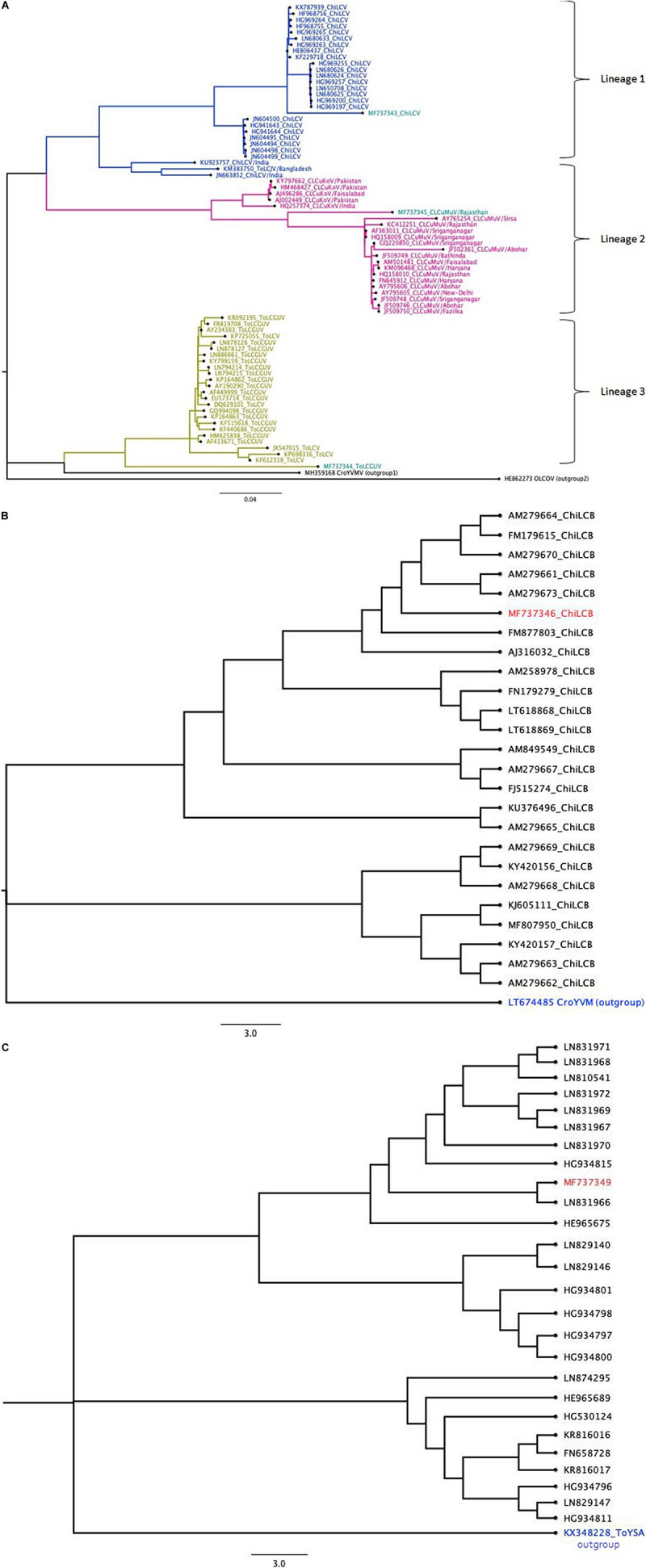
The maximum-likelihood (ML) phylogenetic tree of aligned complete nucleotide sequences of begomovirus isolates obtained from chili; DNA-A of *Chili leaf curl virus*, DNA-A of *Tomato leaf curl Gujarat Virus*, and DNA-A of *Cotton leaf curl Multan virus* **(A)**; *Chili leaf curl betasatellite* **(B)**; *Cotton leaf curl Multan alphasatellite* **(C)**.

To analyze the phylogenetic relationship, evolutionary history was inferred using the ML Tree based on the best fit nucleotide substitution model (Table 3). In a phylogenetic analysis of DNA-A, 3 lineages were observed in which isolate MM1 clustered with lineage 1, isolate CS1 and RV1 exhibited clustering with lineage 2 and 3, respectively (Figure 2A). The highest nucleotide identity and grouping of DNA-A of isolate MM1 (MF737343), with ChiLCV isolates suggests its closeness or ancestry with ChiLCV. Likewise, DNA-A of isolate CS1 had the highest nucleotide identity and phylogeny with ToLCGV suggested its origin from ToLCGV, concomitantly, the ancestry of isolate RV1 from CLCuMuV was also affirmed by its phylogeny and highest nucleotide identity with CLCuMuV. Isolate MM2 of ChiLCB showed clustering with previous ChiLCB isolates from India and Pakistan (Figure 2B). Isolate MM3 of CLCuMuA exhibited common ancestry with other isolates of CLCuMuA reported from Pakistan (Figure 2C).

### Detection of Recombination in ChilCD

To determine the incidence of recombination, RDP analysis was conducted based on alignments with full-length sequences of the top 25 hit retrieved from the NCBI database. We used several algorithms (RDP, GENECONV, CHIMERA, BOOTSCAN, MAXCHI, 3SEQ, and SISTER SCAN) implemented in RDP 4.2 and perceived DNA-A genomic region of CLCuMuA and ToLCGV which has high number of recombination events (Table 4). The βC1 protein of ChiLCB exhibited one recombination breakpoint (876-1219 nucleotide position) with isolate chM52 (AM279670; minor parent) from Pakistan and clone TC241 (KJ605111; major parent) from India (Table 4).

**TABLE 4:** Summary of recombination Breakpoints in begomoviral clones as well its associated satellites, calculated by different algorithms.

Clone Name*	**Event**	**Breakpoint position**		**Putative Minor Parent**	**Putative Major Parent**	**Detection Methodology^$^**	**P value^@^**
		**Begin**	**End**				
MM1	1	540	1209	KF229718 (ChiLCV[OM:12])	Unknown	RGBMCS**3seq**	4.28E−57
CS-1	1	148	2383	Unknown	FR819708 (ToLCGV[PK:11]	RGBMCS**3Seq**	1.12E−51
	2	1615	1975	AF449999 (ToLCGV[IN:99]	Unknown	RMCS**3Seq**	1.03E−25
	3	586	856	KP725055 (ToLCGV[IN:14]	DQ629101 (ToLCGV[IN:09]	GBMCS**3Seq**	3.17E−11
RV-1	1	1610	2170	HQ158009 (CLCuMuV[IN:09]	Unknown	**R**GBMCS3Seq	2.51E−28
	2	2688	496	KC412251 (CLCuMuV[IN:06]	Unknown	**R**GBMCS3Seq	5.55E−21
	3	2282	2687	JF502361 (CLCuMuV[IN:10]	KM383750 (CLCuMuV[BD:07]	RGMC**S**3Seq	5.29E−17
MM-2	1	876	1219	AM279670 (ChiLCB[PK:04]	KJ605111 (ChiLCB[IN:09]	RGBMC**S**3seq	3.69E−18
	2	133	483	Unknown	KJ605111 (ChiLCB[IN:09]	RBMS**3seq**	1.61E−10
MM-3	1	609	901	LN831966 (CLCuMuA[PK:15]	HG934815 (CLCuMuA[PK:13]	**R**MC3Seq	1.80E−04

* MM1, ChiLCV (MF737343); CS-1, ToLCGV(MF737344); RV-1, CLCuMuV(MF737345); MM-2, ChiLCB(MF737346); MM-3, CLCuMuA(MF737349). ^@^The lowest *P*-value calculated for the underline and bold method are given in the column. ^$^R, RDP; G, GeneConv; B, Bootscan; M, MaxChi; C, Chimarea; S, SiScan; 3Seq, Sequence Triplets.

### Population Structure and Substitution Rate Estimation of Begomoviruses and Satellites

From a developmental viewpoint, along with recombination, nucleotide substitution plays a vital role to attain genetic variation and evolution of begomovirus. CLCuMuV was determined to have a high degree of genetic variability (π > 0.08) i.e., 0.0914 (Table 5) and showed to be the most diverse begomovirus (Pagán and García-Arenal, 2018). We also assessed the nucleotide substitution rate of DNA-A genomes of ChiLCV, CLCuMuV, ToLCGV, and associated alphasatellite and betasatellite utilizing the various parameters recorded in Table 5. The mean nucleotide substitution rate for CLCuMuV isolate RV1 was observed to be higher (7.54 × 10^–4^) than ChiLCV and ToLCGV and suggests rapid evolution. Since mutation plays a significant role in selection process leading to genetic variation, we measured the rate of mutation of all the three codon position and found that ChiLCV isolate MM1, CLCuMuV isolate RV1 had higher mutation rate in codon position 1 and high mutation rate was observed in codon position 2 in ToLCGV isolate CS1 (Table 6).

**TABLE 5:** Nucleotide diversity of begomoviruses and its satellite.

Clone Name*	**s**	**η**	**π**	**k**	**θ-w**	**θ – η**
MM1	330	339	0.03002	82.698	0.0267	0.0274
CS-1	666	739	0.0343	91.420	0.0559	0.0620
RV-1	855	1014	0.0914	219.897	0.0941	0.1117
MM-2	414	487	0.0659	87.840	0.0693	0.0816
MM-3	231	251	0.0297	40.415	0.0446	0.0484

* Note-MM1, ChiLCV (MF737343); CS-1, ToLCGV (MF737344); RV-1, CLCuMuV(MF737345); MM-2, ChiLCB(MF737346); MM-3, CLCuMuA(MF737349).

**TABLE 6:** Mean substitution rate and codon position mutation rate for begomovirus and associated satellites clones.

	**MM1**		**CS-1**		**RV-1**		**MM-2**		**MM-3**	
**Mean substitution rate**	**Relaxed clock (ESS value)**	**Strict clock (ESS value)**	**Relaxed (ESS value)**	**Strict (ESS value)**	**Relaxed (ESS value)**	**Strict (ESS value)**	**Relaxed (ESS value)**	**Strict (ESS value)**	**Relaxed (ESS value)**	**Strict (ESS value)**
	7.220E-4 (05)	2.501E-3 (386)	7.472E-4 (137)	3.711E-4 (1103)	1.499E-4 (09)	2.383E-7 (187)	4.208E-4 (07)	6.459E-4 (161)	7.540E-4 (05)	1.773E-6 (90)
at 95% HPD interval	(4.016E-31, 1.892E-3)	(1.626E-3, 3.236E-3)	(2.831E-4, 1.219E-3)	(2.514E-4, 5.054E-4)	(5.043E-59, 1.347E-3)	(3.180E-58, 7.672E-8)	(1.331E-28, 9.771E-4)	(2.838E-4, 9.797E-4)	(2.425E-19, 4.143E-3)	(0.104E-58, 5.289E-7)
CP1 mu	1.4977 (8359)	1.4958 (8457)	0.9385 (8055)	0.8792 (9001)	1.2756 (69)	1.2509 (7910)	0.7726 (8141)	0.7738 (9001)	1.8592 (7503)	1.8956 (7404)
CP2 mu	0.7196 (8230)	0.7197 (8794)	1.2696 (8434)	1.3156 (8947)	0.6942 (1518)	0.7178 (8422)	1.1957 (7946)	1.1955 (8428)	0.6769 (7951)	0.6557 (7614)
CP3 mu	0.7821 (9001)	0.7840 (8705)	0.7919 (7803)	0.8051 (895)	1.0302 (173)	1.0313 (8322)	1.0318 (7579)	1.0307 (8380)	0.4627 (7603)	0.4474 (8543)

* MM1, ChiLCV (MF737343); CS-1, ToLCGV(MF737344); RV-1, CLCuMuV(MF737345); MM-2, ChiLCB(MF737346); MM-3, CLCuMuA(MF737349).

Additionally, haplotype sequence polymorphisms and diversity are also studied for ChiLCV based on host (Table 7). Regarding haplotype distributions of ChiLCV, a total of 62 haplotypes were detected out of the 69 sequences analyzed. The highest number of haplotypes was observed in Chili with 37 haplotypes. All the subpopulations recorded high haplotype diversity (hd > 0.95). The number of segregating sites was highest in Chili with 1165 and nearly 212 average nucleotide differences. A high degree of nucleotide diversity (0.14) with an average of 1072 nucleotide differences was noted in the other hosts (Table 7).

**TABLE 7:** Descriptive genetic parameters of diversity of *Chili leaf curl virus* based on different hosts.

**Host**	**No. of seq**	**S**	**Eta**	**Hd**	**K**	**π**	**h**	**θ-w**	**θ-Eta**
Chili	43	1165	1527	0.98	212	0.07	37	0.1	0.13
Papaya	9	849	1044	1	367	0.13	9	0.12	0.14
Tomato	8	533	630	0.96	199	0.07	7	0.07	0.08
others	9	855	1072	1	402	0.14	9	0.12	0.14
Total	69	1426	2074	0.99	257	0.09	62	0.11	0.16

### Agro-Infectivity of Cloned DNA

**FIGURE 3:**
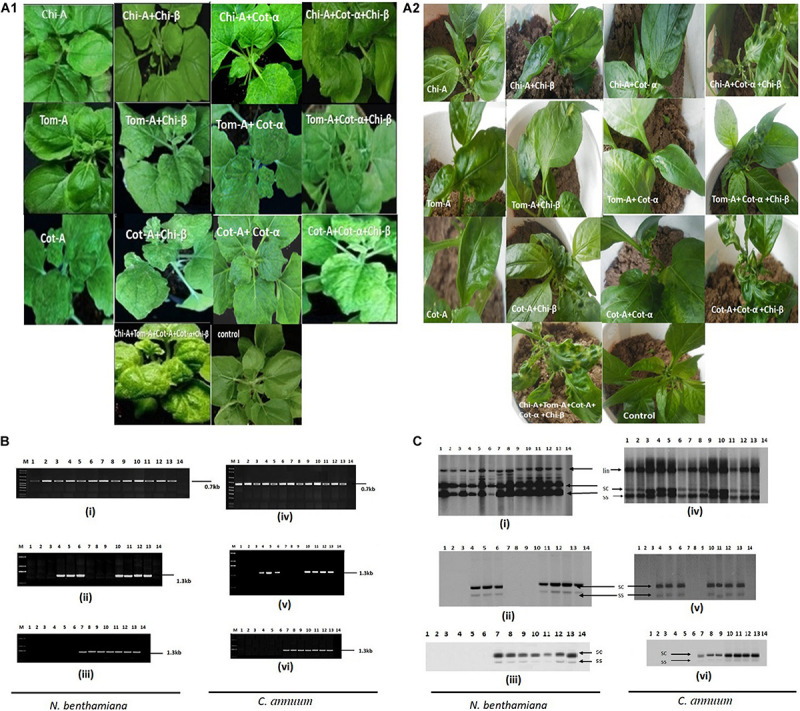
Infectivity and viral DNA accumulation analysis. Symptoms induction in *N. benthamiana* **(A1)** and chili **(A2)** plants infected with various begomoviruses alone and with their respective cognate satellite as indicated. **(B)** PCR amplification of various begomovirus and betasatellite based on infectivity assay in *N. benthamiana* and chili plants: **(i,iv)** A product of 0.7 kb amplified by using CP primers AC-1048/AV-494 (Wyatt, 1996); **(ii,v)** 1.3 kb amplified product by primer beta01/beta02 (Briddon et al., 2002); and **(iii,vi)** 1.3 kb amplified product by primer set UN101/UN102 (Bull et al., 2003). **(C)** Respective DNA accumulation of various begomovirus and betasatellite are analyzed by Southern hybridization in *N. benthamiana* **(i–iii)** and chili **(iv–vi)** using specific probes. The lane description of the gel and blot are as follows: M, marker; lane 1, Cot-A; lane 2, Chi-A; lane 3, Tom-A; lane 4, Chi-A + Chi-β; lane 5, Cot-A + Chi-β; lane 6, Tom-A + Chi-β; lane 7, Chi-A + Cot-α; lane 8, Cot-A + Cot-α; lane 9, Tom-A + Cot-α; lane 10, Tom-A + Chi-β + Cot-α; lane 11, Cot-A + Chi-β + Cot-α; lane 12, Chi-A + Chi-β + Cot-α; lane 13, Chi-A + Tom-A + Cot-A + Chi-β + Cot-α; and lane 14, control.

Typical begomovirus symptoms including leaf curling, leaf crumpling and yellowing were observed in infiltrated *N. benthamiana* and Chili plants after 21 dpi (Table 8 and Figure 3A1,A2). Plants were infiltrated with various combinations of DNA-A and satellite molecules. The credibility of the infectivity test was affirmed by PCR using coat protein-specific primer set (AC-1048/AV-494) and begomovirus DNA was identified in many of the plants infiltrated with pCAMBIA-A-DNA and showed that tested begomoviruses are the causative agents of ChiLCD. To check the associated satellite molecules, isolated DNA was amplified using a universal primer for DNA-α and DNA-β (Briddon et al., 2002) and observed the same thermal profile as for DNA-A (Figure 3B). Our results demonstrated that ChiLCB, are very flexible in their interactions with new non-cognate helper viruses. The infectious clone of DNA-A, upon agroinfiltration in plants, developed mild symptoms. It demonstrated that three begomovirus species alone could infect *N. benthamiana* and chili, turning mild symptoms (Qadir et al., 2019; Sattar et al., 2019). However, co-inoculation with betasatellite, symptoms of severe leaf curling, downward curling and vein yellowing were observed (Table 8 and Figure 3C). Our results also indicate that CLCuMuA had unable to show any effective symptoms as induced by DNA-A or its complex with betasatellite (Luo et al., 2019). The infection of *N. benthamiana* and chili plants was confirmed by southern blot hybridization (Figure 3C).

**TABLE 8:** Infectivity of cloned viral components in *N. benthamiana*.

**Sr. No.**	**Viral construct^#^**	**No. of symptomatic plants/no. of inoculated plants (*N. benthamiana*)**	**Incubation period in days**	**Symptoms in inoculated plants^@^**	**No. of symptomatic plants/no. of inoculated plants (Chili)**	**Incubation period in days**	**Symptoms in inoculated plants^@^**	**PCR**		
								**DNA-A**	**DNA-β**	**DNA-α**
	Chi-A	9/10	21	MLC	10/10	20	MLC	+	-	-
	Chi-A + Chi-β	10/10	17	SLC	9/10	21	DLC	+	+	-
	Chi-A + Cot α	8/10	20	MLC	9/10	18	MLC	+	-	+
	Chi-A + Chi-β + Cot-α	9/10	18	MLC	10/10	20	SLC	+	+	+
	Tom-A	9/10	20	LC	8/10	19	LC	+	-	-
	Tom-A + Chi-β	10/10	21	DLC	10/10	19	DLC	+	+	-
	Tom-A + Cot α	9/10	19	MLC	8/10	18	MLY	+	-	+
	Tom- A + Chi-β + Cot-α	10/10	21	DLC, SLC	10/10	17	SLC	+	+	+
	Cot-A	9/10	19	LC	10/10	21	LC	+	-	-
	Cot-A + Chi-β	10/10	18	LC	9/10	21	DLC,MLY	+	+	-
	Cot-A + Cot α	8/10	20	DLC	9/10	20	LC	+	-	+
	Cot-A + Chi-β + Cot-α	10/10	20	MLY, LC	10/10	19	SLC,MLY	+	+	+
	Chi-A + Tom-A + Cot-A + Chi-β + Cot-α	10/10	21	LC, LY, DLC	10/10	20	SLC,MLY,DLC	+	+	+
	pCAMBIA	0/10	21	No symptoms	0/10	21			-	-

*^#^Chi-A, MM1 ChiLCV (MF737343); Tom-A, CS-1 ToLCGV (MF737344); Cot-A, RV-1 CLCuMuV (MF737345); Chiβ, MM-2 ChiLCB (MF737346); Cot α, MM-3 CLCuMuA (MF737349). ^@^MLC, mild leaf curling; LC, leaf curling; SLC, severe leaf curling; DLC, downward leaf curling; MLY, mild leaf yellowing; LY, leaf yellowing.*

## Discussion

Chili is an important field crop grown in Rajasthan Province and infection with begomoviruses has a high agronomic impact by inhibiting plant growth and reduced fruit quality. Chili accounts for 20–30% of total Indian spices exports valuing approx Rs. 400-500 crores. In India, *Chili leaf curl virus* (ChiLCV), *Chili leaf curl India virus* (ChiLCINV), *Chili leaf curl Kanpur virus* (ChiLCKaV), *Chili Leaf curl Vellanad virus* (ChiLCVV), *Pepper leaf curl Bangladesh virus* (PepLCBV), *Tomato leaf curl New Delhi virus* (ToLCNDV), *Papaya leaf curl virus* (PaLCuV), *Radish leaf curl virus* (RaLCuV), *Tomato leaf curl virus* (ToLCV) and *Tomato leaf curl Joydebpur virus* (ToLCJV) have been reported in chili crop, thereby causing leaf curl disease (Malathi et al., 2017). Mixed infections have been reported frequently, in some well-studied crops but so far there are no reports have been found on the association of ChiLCV, ToLCGV, and CLCuMuV with leaf curl disease of chili. We reported in this study the occurrence of three distinct begomoviruses such as ChiLCV, ToLCGV, CLCuMuV and their satellite molecules in chili. The high degree of genetic variability was observed in the genome of ToLCGV as compared with isolates of ChiLCV and CLCuMuV. These mixed infections are the prerequisite of recombination resulting a diversification largely exploited by begomoviruses (Morilla et al., 2004).

Furthermore, the dN/dS ratio of DNA-A of ToLCGV and CLCuMuV was higher than that of ChiLCV. These results suggest that ToLCGV is evolving under more relaxed negative selection than ChiLCV and CLCuMuV. The presence of lesser nucleotide sequence divergence was observed in alphasatellite molecule and less recombination among them was also evident which implies that the alphasatellite molecules are under robust evolutionary limitation than other DNA (Lima et al., 2017). Computing the transitions/transversions bias (R) is very substantial for correct inference of phylogeny, divergence time estimation and for understanding the evolution of genomes (Yang and Yoder, 1999; Lyons and Lauring, 2017) as it allows us to validate the presence of bias in the nucleotide substitutions. In our datasets, the transitions/transversions bias (R) is greater to or equal to one was consistent with the principle that transitions were more frequent than transversions in analyzed sequences. Phylogenetic analysis revealed that CLCuMuV and ToLCGV as well as betasatellite and alphasatellite molecules grouped with other Indian and Pakistani isolates, whereas ChiLCV showed grouping with the isolates reported from Oman. Higher negative Tajima values of DNA-A of ToLCGV and CLCuMuA implies an excess of low-frequency polymorphisms, concomitantly the Tajima D value near to zero of DNA-A of ChiLCV showed neutral selection in analyzed sequences, suggesting different ancestry and origin of the isolates (Hill and Unckless, 2017).

Emergence of new begomoviruses relies on how frequently begomoviruses recombine with each other and eventually acquire satellite molecules (Juárez et al., 2019). Begomoviruses not only recombine with each other but they can also experience recombination with satellite molecules or DNA-B (Nawaz-ul-Rehman and Fauquet, 2009; Venkataravanappa et al., 2011). We observed a high mutation rate in the CP region and the Rep region of CLCuMuV and ToLCGV, strengthen the previous results obtained by Saleem et al. (2016). Data of this study suggest that ChiLCV, ToLCGV, and CLCuMuV have intricate recombination history with significant contribution of interspecific recombination. Similar to RNA viruses, geminiviruses are known to have a high nucleotide substitution rate (Duffy and Holmes, 2008). Higher genetic variability was observed in begomoviruses concomitant to ChiLCD than other begomoviruses (Melgarejo et al., 2013). These begomoviruses have non-uniformly distributed nucleotide diversity in their genome (Lima et al., 2013). In concurrence with different studies, our results accentuate that the major evolutionary factor which leads to the emergence of these begomoviruses and associated satellite molecules is purifying selection (Silva et al., 2012; Ho et al., 2014).

Several reports such as geminiviruses infecting cassava in Africa (Sangaré et al., 2001), suggest that mixed infections are the potential cause of geminivirus variability due to recombination events (Rentería-Canett et al., 2011). Mixed virus infection complex is affected by location, growing season, presence of bridging crops and biotype of vector. Mixed virus infections are significant to virus evolution because they provide the precondition for recombination, which may contribute to the appearance of more severe virus strains or new begomovirus species (Ribeiro et al., 2003; Ala-Poikela et al., 2005; Kumar, 2019). Several studies reported the mixed infection of begomoviruses in various crops worldwide, such as mixed infection of begomoviruses infecting tomato, pepper and cucurbit crops in Nicaragua (Ala-Poikela et al., 2005), *Pepper huasteco yellow vein virus* (PHYVV) and *Pepper golden mosaic virus* (PepGMV) on pepper in Mexico (Ala-Poikela et al., 2005), an association of *Tomato leaf curl New Delhi virus*, *Squash leaf curl China virus* and *Tomato leaf curl Palampur virus* infecting pumpkins in India (Jaiswal et al., 2012). In India, the most prevalent betasatellites associated with ChiLCD have been reported from tomato and chili rather than from weeds (Kumar Y. et al., 2011). These reports suggest that mixed infection is the major influential force in the emergence and spread of begomoviruses. However, the significance of such association needs to be explored for the management of ChiLCD needs to be explored.

The diverse Indian climate supports the year-round cultivation of various crops and the survival of whiteflies. This is the reason a huge number of begomoviruses have been reported in India. The polyphagous nature of vector whitefly and mixed cropping system of India is responsible for its overlapping host range. Earlier studies have demonstrated the ChiLCV infecting variety of crops like tobacco, tomato, papaya, eggplant, petunia (Nehra and Gaur, 2015). CLCMuV was also isolated from infected cotton and hibiscus plants (Malathi et al., 2017). ToLCGV is one of the most predominant and destructive species occurring throughout India and causing severe leaf curl disease of tomato. Here, we reported the association of ChiLCV, ToLCGV and CLCuMuV infection together in chili with more severe disease could be due to synergistic interaction among viruses. Taken together, the results of this study show the ChiLCD complex is in an evolving nature as a result of infection of multiple begomoviruses to overcome resistance and to expand its host range by recombination, mutation, and virus capturing. This means that the expanded frequency and extension in the host range of begomovirus disease complexes can posture a genuine future threat to agriculture practices in India. Chili crop diseases are of great concern to farmers as the severity of the disease has increased in the last few years. A further study of the evolving nature of begomoviruses at Rajasthan and surrounding areas will help to understand the ChiLCD complex in a broader sense and incorporation of new strategies to control complex diseases in chili.

## Data Availability Statement

The datasets generated for this study can be found in NCBI, accession numbers MF737343, MF737344, MF737345, MF737346, and MF737349.

## Author Contributions

RG and PS designed and analyzed the data and wrote and edited the first draft. MM and RV contributed equally to the experiments and computational work. AM helped in computational analysis. All authors read and approved the manuscript for submission.

## Conflict of Interest

The authors declare that the research was conducted in the absence of any commercial or financial relationships that could be construed as a potential conflict of interest.

## Footnotes

1 http://www.ncbi.nlm.nih.gov/projects/gorf/

2 http://beast.bio.ed.ac.uk/Tracer

## Supplementary Material

Supplementary Figure 1 Genome Organization of *Chili leaf curl virus*: ChiLCV (MF737343); *Tomato leaf curl Gujarat virus:* ToLCGV (MF737344); *Cotton leaf curl Multan virus*: CLCuMuV (MF737345); *Chili leaf curl betasatellite*: ChiLCB (MF737346) and *Cotton leaf curl Multan alphasatellite*: CLCuMuA (MF737349).

Supplementary Figure 2 Color pairwise nucleotide identity matrix of three begomovirus species and two satellites. **(A)** *Chili leaf curl virus*: ChiLCV (MF737343); **(B)** *Tomato leaf curl Gujarat virus*: ToLCGV (MF737344); **(C)** *Cotton leaf curl Multan virus*: CLCuMuV (MF737345); *Chili leaf curl betasatellite*: **(D)** ChiLCB (MF737346) and **(E)** *Cotton leaf curl Multan alphasatellite*: CLCuMuA (MF737349) are described in the study and other sequences were retrieved from NCBI database.

Supplementary Table 1 Sequences used for *Chili leaf curl virus*: ChiLCV (MF737343) studies.

Supplementary Table 2 Sequences used for *Chili leaf curl betasatellite*: ChiLCB (MF737346) studies.

Supplementary Table 3 Sequences used for *Cotton leaf curl Multan virus*: CLCuMuV (MF737345) studies.

Supplementary Table 4 Sequences used for *Cotton leaf curl Multan alphasatellite*: CLCuMuA (MF737349) studies.

Supplementary Table 5 Sequences used for *Tomato leaf curl Gujarat virus*: ToLCGV (MF737344) studies.
