# Supplementary figures and images for: Biology and Interaction of the Natural Occurrence of Distinct Monopartite Begomoviruses Associated With Satellites in Capsicum annum From India
Source: Front Microbiol. 2020 Oct 7;11:512957. doi: 10.3389/fmicb.2020.512957 (PMC7575687; doi:10.3389/fmicb.2020.512957)

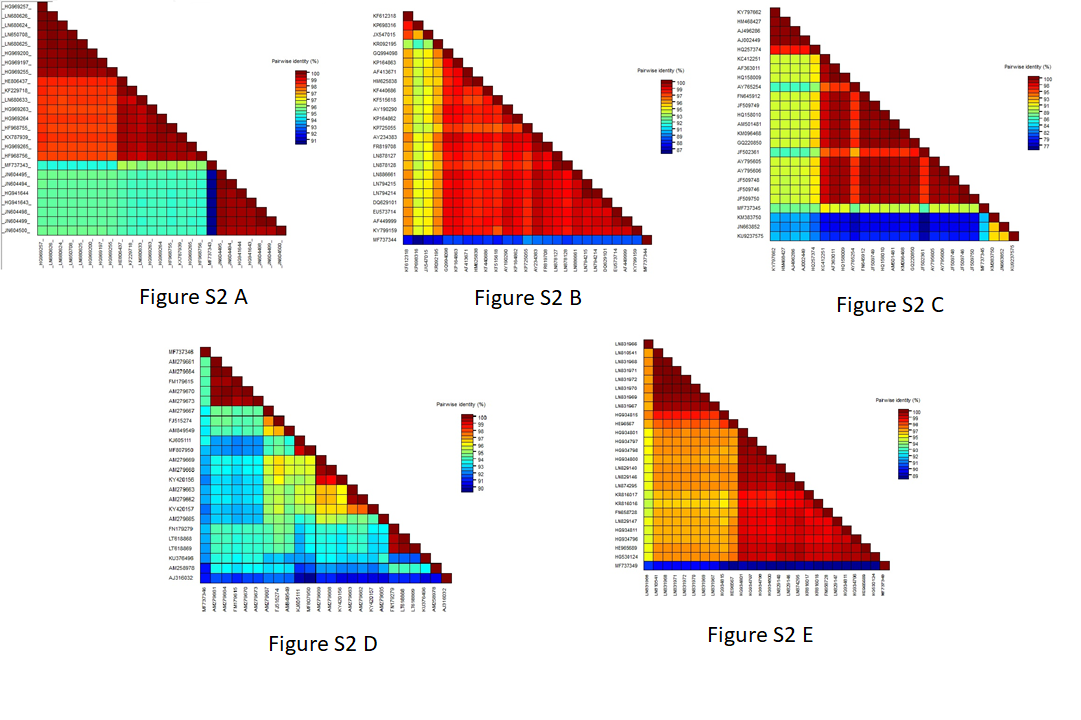

Supplement: Supplementary Figure 1 — Genome Organization of Chili leaf curl virus: ChiLCV (MF737343); Tomato leaf curl Gujarat virus: ToLCGV (MF737344); Cotton leaf curl Multan virus: CLCuMuV (MF737345); Chili leaf curl betasatellite: ChiLCB (MF737346) and Cotton leaf curl Multan alphasatellite: CLCuMuA (MF737349). [file Image_2.PNG]

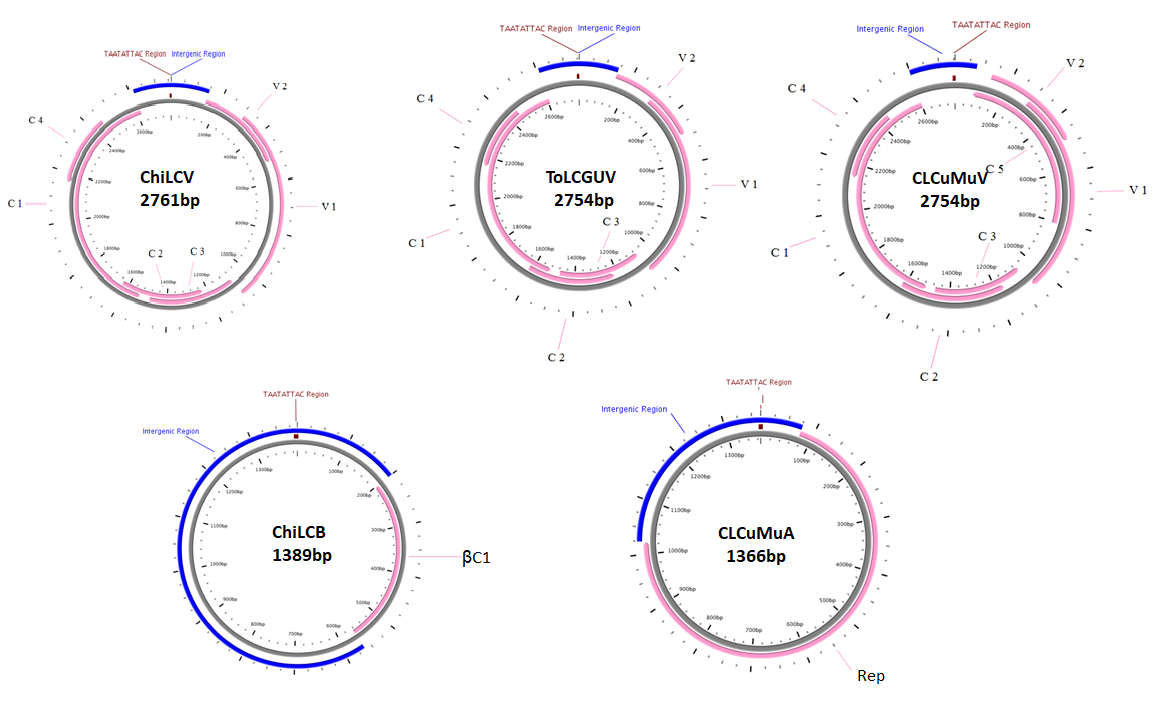

Supplement: Supplementary Figure 2 — Color pairwise nucleotide identity matrix of three begomovirus species and two satellites. (A) Chili leaf curl virus: ChiLCV (MF737343); (B) Tomato leaf curl Gujarat virus: ToLCGV (MF737344); (C) Cotton leaf curl Multan virus: CLCuMuV (MF737345); Chili leaf curl betasatellite: (D) ChiLCB (MF737346) and (E) Cotton leaf curl Multan alphasatellite: CLCuMuA (MF737349) are described in the study and other sequences were retrieved from NCBI database. [file Image_1.PNG]
