# Supplementary material for: Biology and Interaction of the Natural Occurrence of Distinct Monopartite Begomoviruses Associated With Satellites in Capsicum annum From India
Source: Front Microbiol. 2020 Oct 7;11:512957. doi: 10.3389/fmicb.2020.512957 (PMC7575687; doi:10.3389/fmicb.2020.512957)
Supplement: Supplementary Table 1 — Sequences used for Chili leaf curl virus: ChiLCV (MF737343) studies. [file Data_Sheet_1.docx]

**Supplementary tables (S)**

**Table S1: Sequences used for *Chilli leaf curl virus*: ChiLCV (MF737343) studies**

| Accession no. | Year | Host | Country |
| --- | --- | --- | --- |
| HE806437 | 2011 | Tomato | Oman |
| KX787939 | 2015 | *Citrullus lanatus* | Oman |
| HG969264 | 2013 | *Citrullus lanatus* | Oman |
| HG969265 | 2013 | - | Oman |
| HG969263 | 2013 | Radish | Oman |
| KF229718 | 2012 | Tomato | Oman |
| HF968755 | 2012 | petunia spp. | Oman |
| HF968756 | 2012 | Petunia spp. | Oman |
| LN680633 | 2014 | Tomato | Oman |
| HG969257 | 2013 | - | Oman |
| LN650708 | 2014 | Tomato | Oman |
| LN680625 | 2014 | Tomato | Oman |
| LN680626 | 2014 | Tomato | Oman |
| HG969200 | 2013 | Tomato | Oman |
| HG969197 | 2013 | Tomato | Oman |
| LN680624 | 2014 | Tomato | Oman |
| HG969255 | 2013 | - | Oman |
| JN604495 | 2011 | Tomato | Oman |
| JN604494 | 2011 | Tomato | Oman |
| HG941644 | 2013 | Tobacco | Oman |
| JN604498 | 2011 | Tomato | Oman |
| JN604499 | 2011 | Tomato | Oman |
| HG941643 | 2013 | Basil | Oman |
| JN604500 | 2011 | Tomato | Oman |

**Table S2: Sequences used for *Tomato leaf curl Gujarat virus:*** ToLCGUV **(MF737344) studies**

| Accession no. | Year | Host | Country |
| --- | --- | --- | --- |
| KP725055 | 2014 | papaya | India |
| AY234383 | 2000 | tomato | Nepal |
| LN886661 | 2014 | tomato | Pakistan |
| FR819708 | 2011 | *Xanthium strumarium* | Pakistan |
| LN794215 | 2013 | cotton | Pakistan |
| LN794214 | 2013 | cotton | Pakistan |
| EU573714 | 2008 | tomato | India |
| AF449999 | 1999 | tomato | India |
| DQ629101 | 2009 | tomato | India |
| KP164863 | 2007 | tomato | India |
| LN878127 | 2015 | tomato | Pakistan |
| AY190290 | 2002 | tomato | India |
| LN878128 | 2015 | tomato | Pakistan |
| KP164862 | 2007 | tomato | India |
| GQ994098 | 2007 | tomato | India |
| KF440686 | 2012 | *Phaseolus vulgaris* | India |
| AF413671 | 1999 | tomato | India |
| HM625838 | 2008 | tomato | India |
| KY799159 | 2017 | chilli | Pakistan |
| KF612318 | 2012 | tomato | India |
| JX547015 | 2012 | tomato | India |
| KR092195 | 2012 | tomato | India |
| KF515618 | 2012 | tomato | India |
| KP698316 | 2014 | Ocimum | India |

**Table S3: Sequences used for *Cotton leaf curl Multan virus*: CLCuMuV (MF737345) studies**

| Accession no. | Year | Host | Country |
| --- | --- | --- | --- |
| KC412251 | 2006 | cotton | India |
| AF363011 | 2001 | cotton | India |
| FN645912 | 2007 | cotton | India |
| AY795605 | 2003 | - | India |
| HQ158010 | 2006 | cotton | India |
| JF509748 | 2005 | cotton | India |
| AY795606 | 2003 | - | India |
| JF509749 | 2005 | cotton | India |
| JF509746 | 2003 | cotton | India |
| AM501481 | 2005 | tomato | Pakistan |
| GQ220850 | 2008 | cotton | India |
| KM096468 | 2013 | cotton | India |
| KY797662 | 2016 | jasminum | Pakistan |
| AJ002449 | 1998 | okra | Pakistan |
| AJ496286 | 2003 | cotton | Pakistan |
| HM468427 | 2008 | *Gossypium stocksii* | Pakistan |
| HQ257374 | 2010 | Cotton | India |
| AY765254 | 2004 | - | India |
| JF502361 | 2010 | cotton | India |
| KM383750 | 2007 | tomato | Bangladesh |
| JN663852 | 2010 | chilli | India |
| KU923757 | 2015 | chilli | India |
| JF509750 | 2005 | cotton | India |
| HQ158009 | 2009 | cotton | India |

**Table S4: Sequences used for *Chilli leaf curl betasatellite*: ChiLCB (MF737346) studies**

| Accession no. | Year | Host | Country |
| --- | --- | --- | --- |
| AM279661 | 2004 | chilli | Pakistan |
| AM279664 | 2004 | chilli | Pakistan |
| AM279673 | 2004 | chilli | Pakistan |
| FM179615 | 2009 | *Solanum tuberosum* | Pakistan |
| AM279667 | 2004 | chilli | Pakistan |
| FJ515274 | 2006 | *N. bentamiana* | Pakistan |
| AM279670 | 2004 | chilli | Pakistan |
| FN179279 | 2009 | chilli | Pakistan |
| LT618868 | 2016 | tomato | Pakistan |
| LT618869 | 2016 | tomato |  |
| AM849549 | 2006 | chilli | Pakistan |
| KJ605111 | 2009 | tomato | India |
| MF807950 | 2017 | tomato | India |
| KU376496 | 2015 | chilli | India |
| AM279665 | 2004 | chilli | Pakistan |
| AM279669 | 2004 | chilli | Pakistan |
| AM279668 | 2004 | chilli | Pakistan |
| AM279663 | 2004 | chilli | Pakistan |
| AM279662 | 2004 | chilli | Pakistan |
| KY420156 | 2015 | cotton | Pakistan |
| AM258978 | 2004 | chilli | Pakistan |
| KY420157 | 2015 | cotton | Pakistan |
| AJ316032 | 2003 | chilli | Pakistan |

**Table S5: Sequences used for *Cotton leaf curl Multan alphasatellite*: CLCuMuA (MF737349) studies**

| Accession no. | Year | Host | Country |
| --- | --- | --- | --- |
| LN831966 | 2015 | cotton | Pakistan |
| LN810541 | 2015 | cotton | Pakistan |
| LN831971 | 2015 | cotton | Pakistan |
| LN831968 | 2015 | cotton | Pakistan |
| LN831970 | 2015 | cotton | Pakistan |
| LN831972 | 2015 | cotton | Pakistan |
| LN831969 | 2015 | cotton | Pakistan |
| LN831967 | 2015 | cotton | Pakistan |
| HG934801 | 2013 | cotton | Pakistan |
| HG934797 | 2013 | cotton | Pakistan |
| HG934815 | 2013 | cotton | Pakistan |
| KR816017 | 2011 | cotton | Pakistan |
| KR816016 | 2010 | Cotton | Pakistan |
| LN874295 | 2013 | cotton | Pakistan |
| HG934798 | 2013 | cotton | Pakistan |
| LN829140 | 2015 | cotton | Pakistan |
| HG934800 | 2013 | cotton | Pakistan |
| LN829147 | 2015 | cotton | Pakistan |
| FN658728 | 2007 | cotton | India |
| HG934796 | 2013 | cotton | Pakistan |
| LN829146 | 2015 | cotton | Pakistan |
| HG530124 | 2012 | cotton | Pakistan |
| HE965689 | 2011 | cotton | Pakistan |
| HG934811 | 2013 | cotton | Pakistan |
| HE965675 | 2011 | cotton | Pakistan |
